# Nodal and osseous oligometastatic prostate cancer: a cohort including the introduction of PSMA-PET/CT-guided stereotactic and hypofractionated radiotherapy with elective nodal therapy

**DOI:** 10.1007/s00432-022-04229-1

**Published:** 2022-08-27

**Authors:** Ahmed Gawish, Matthias Walke, Burkard Röllich, Hans-Joachim Ochel, Thomas B. Brunner

**Affiliations:** 1 Department of Radiation Oncology, University Hospital Magdeburg, Leipziger Str. 44, 39120 Magdeburg, DE Germany; 2 Department of Radiation Oncology, Medical University of Graz, 8036 Graz, Austria

**Keywords:** SBRT, HFRT, Salvage, Prostata cancer, PSMA-PET, ENRT

## Abstract

**Purpose:**

Oligometastatic prostate cancer is heavily investigated, and conventionally fractionated elective nodal treatment appears to increase biochemical relapse-free (bRFS) survival. The novelty of this report is to present elective nodal radiotherapy (ENRT) with simultaneous integrated boost with stereotactic (SBRT) or hypofractionated radiotherapy (HoFRT) for tolerance and for bRFS which we compared with SBRT of the involved field (IF) only.

**Materials and methods:**

Patients between 2018 and 2021 with and oligometastatic prostate cancer treated with SBRT or hypofractionation were eligible. A radiobiologically calculated simultaneous integrated boost approach enabled to encompass elective nodal radiotherapy (ENRT) with high doses to PSMA-positive nodes. A second group had only involved field (IF) nodal SBRT.

**Results:**

A total of 44 patients with 80 lesions of initially intermediate- (52%) or high-risk (48%) D’Amico omPC were treated with SBRT to all visible PSMA-PET/CT lesions and 100% of the treated lesions were locally controlled after a median follow-up was 18 months (range 3–42 months). Most lesions (56/80; 70%) were nodal and the remainder osseous. Median bPFS was 16 months and ADT-free bPFS 18 months. ENRT (31 patients) versus IF (13 patients) prevented regional relapse more successfully. At univariate analysis, both initial PSA and length of the interval between primary diagnosis and biochemical failure were significant for biochemical control. Treatment was well tolerated and only two patients had toxicity ≥ grade 3 (1 GU and 1 GI, each).

**Discussion/conclusion:**

SBRT and hypofractionated radiotherapy at curative doses with ENRT was more effective to delay ADT than IF, controlled all treated lesions and was well tolerated.

## Introduction

Oligometastatic prostate cancer is a catch-all word that refers to at least two separate entities with probable unique biochemical signatures and behaviors. Oligorecurrent prostate cancer refers to the development of limited distant sites of dissemination following primary radical prostatectomy (RP) or radiotherapy (RT), whereas de novo oligometastasis refers to a distinct group of patients with prostate cancer that has spread to limited areas prior to receiving definitive therapy. As a result, viable treatment methods for de novo oligometastases must take the complete source tumor into account in addition to distant lesions. A third form, dubbed oligoprogression, is also developing. It refers to individuals who have extensive metastases but have just a few sites of progression on systemic treatment (Cheung 2016; Franzese et al. 2018; Triggiani et al. 2017). Notably, oligoprogressive illness was underrepresented in retrospective investigations, despite the fact that series with mixed histologies (Pembroke et al. 2018), and prostate cancer in particular (Franzese et al. 2018; Triggiani et al. 2017), have revealed a poorer prognosis for individuals with typical oligometastasis.

Typically, individuals with oligometastatic illness were diagnosed using a specified criteria for the number of distant locations implicated. Recent research, however, indicates that individuals with oligometastases have a better prognosis. Tree et al. postulated that treating oligometastases might increase PFS (progression-free survival) by 2–4 years in 20%–40% of patients with various types of cancer, including prostate carcinoma (Tree et al. 2013). There is currently no agreement on the number of nodal or bone lesions that define the "oligo" stage except the ESTRO-ASTRO consensus (Lievens et al. 2020), Lievens and colleagues defined the Oligometastic disease (OMD) can to date be defined as 1–5 metastatic lesions, a controlled primary tumor being optional, but where all metastatic sites must be safely treatable.

Feasibility findings on metastasis-directed treatment for oligorecurrences without ADT indicate that S^2018^BRT (stereotactic body radiation) has a low toxicity rate (Ost et al. 2016; Kneebone et al. 2018; Siva et al. ). Prospective findings using choline or sodium fluoride PET (Positron Emission Tomography) SBRT (Ost et al. 2016; Kneebone et al. 2018) suggest an increase in PFS and a delay in the requirement for ADT when compared to observation alone (Ost et al. 2018). However, in the PSMA-PET era, Kneebone et al. demonstrated conflicting results, with distant recurrences occurring in the majority of patients within 15 months of follow-up (Kneebone et al. 2018). Considering this controversy regarding the impact of local therapy in limited stage IV disease, we chose to conduct a retrospective analysis of patients who received ablative RT (radiotherapy) for < five macroscopic oligometastases (rcN1/cM1).

The purpose of this research was to determine the effectiveness and safety of stereotactic body radiotherapy (SBRT/IGRT) in patients with oligometastatic disease who had a biochemical recurrence following prior surgery. We are the first to describe a combined approach of ENRT with SIB using non-conventional single doses but exclusively SBRT or a hypofractionated fractionation schedule.

## Materials and methods

### Patient selection

**Fig. 1:**
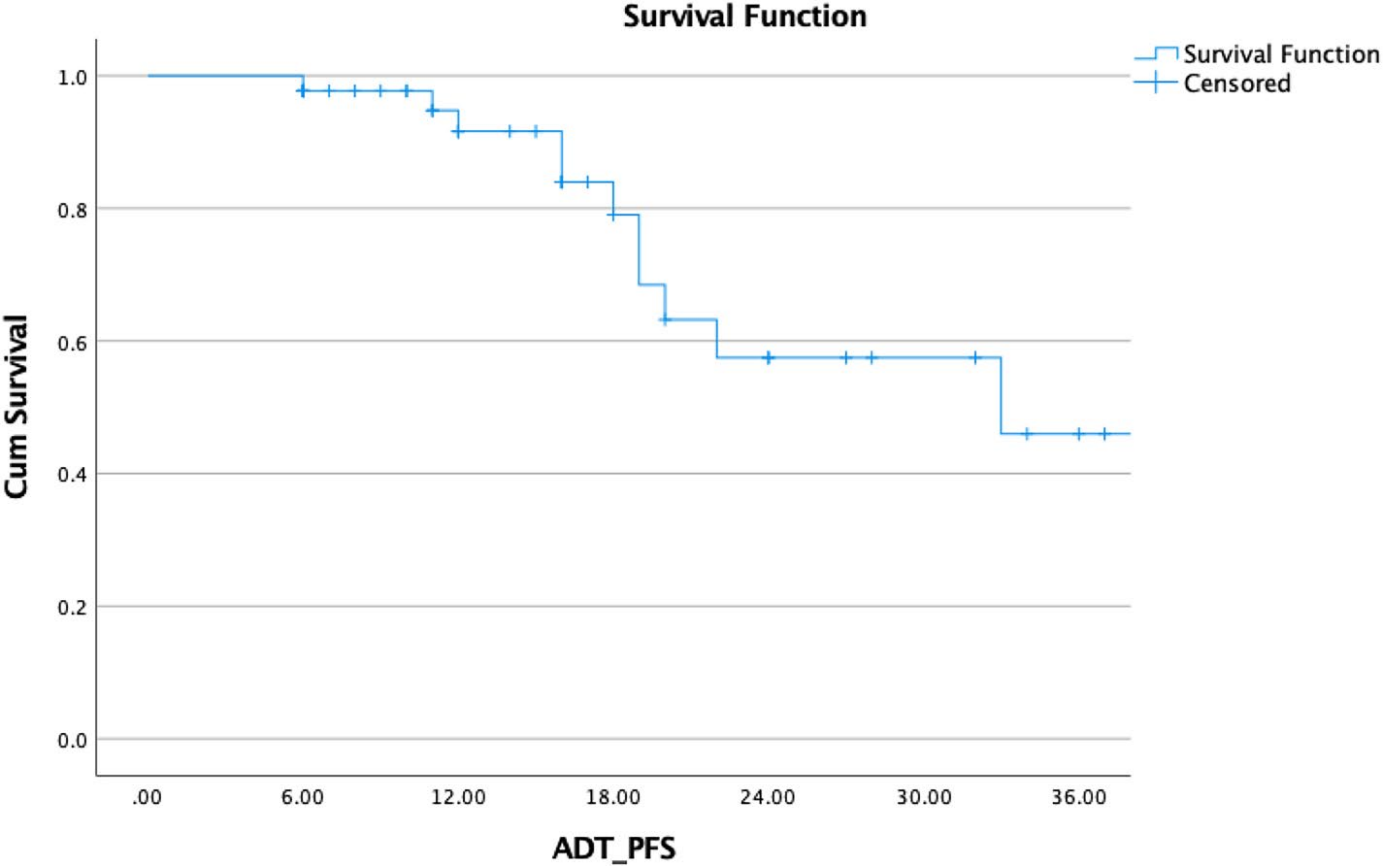
ADT-free_biochemical PFS in patients with oligometastatic prostate cancer

Patients treated between March 2018 and October 2021 at the Department of Radiation Oncology at the University Hospital Magdeburg in Germany with SBRT or hypofractionated radiotherapy of ≥ 3 Gy per fraction for oligometastatic lymph nodes or bone metastases were included in this retrospective analysis. Only patients with less than or equal to five metastases at the time of SBRT were included in the study. Many published studies including an international clinical trial (Lievens et al. 2020; Palma et al. 2012; Milano et al. 2008) have defined oligometastatic disease as having less than or equal to five metastases, and this criterion was utilized in our analysis. Based on the PSMA-PET/CT scan, the diagnosis of oligometastatic illness was confirmed (Fig. 1).

Patients were included in the study if they were above the age of 18, and if they had an Eastern Cooperative Oncology Group (ECOG) score of 2 or less. Repeated SBRT to the same location or verified cerebral metastases were exclusion criteria from the study. SBRT was confirmed and agreed choice of therapy in all cases that were assessed by a multidisciplinary board. The institutional ethics committee gave its support to this study.

### Treatment and follow-up

Patients were immobilized with a vacuum bag and scanned with their arms raised in a supine posture. For treatment of the upper thoracic and cervical areas, additional thermoplastic upper body or head and neck masks were employed. There were no fiducials used. All subjects in the simulation underwent a conventional CT scan with IV contrast and oral contrast if indicated. The treating radiation oncologist decided whether to do an extra 4DCT scan on lymph nodes that were located in position that could not be excluded to be moving with respiration during treatment. Baseline diagnostic PSMA-PET/CT was either performed in planning position or they were fused with planning CT images to the region of interest for tumor delineation. CT simulation and/or PSMA-PET/CT scans were used to determine the gross tumor volume (GTV). No additional CTV margins were added to the GTV. The respective GTVs shown on all phases of the 4DCT scan were aggregated to form an internal target volume (ITV) in circumstances when 4DCT simulation was used. An isotropic margin of 4 mm (mm) was added to the ITV to produce the planning target volume (PTV). An isotropic margin of 4 mm on the CTV was utilized to determine the PTV for patients who did not get a 4DCT simulation scan and for CTVs of elective nodal regions as well as vertebral bodies. IMRT plans were used for all patients.

The Ray-Station™ (Stockholm, Sweden) software and TomoTherapy (Accuray, Sunnyvale, CA, USA) software were used to optimize treatment programs. Different SBRT regimens were utilized depending on the target dimension or metastases location. When dose restrictions for organs at risk were not satisfactory, either replanning or a more protracted fractionation schedule was considered. Generally, hypofractionation and not SBRT was chosen if OAR constraints could not be respected with SBRT and if the OARs of the prostatic fossa were part of the treatment volume. Using daily CBCT, all the patients underwent daily IGRT to ensure the delivery of the right doses to the target volume and to avoid the overdose for OAR.

After radiation therapy, patients were followed up by radiation oncologists every 2–4 months. PSMA-PET/CT, MRI, or CT were all used for routine follow-up imaging.

The local tumor response was evaluated using RECIST (Endpoints Response Evaluation Criteria in Solid Tumors; Eisenhauer et al. 2009). The primary endpoints for oligorecurrent PC were distant progression-free survival (DPFS, defined as the time from the first day of SBRT to the detection of clinical disease outside the PTV following further biochemical progression) and ADT-free survival (ADT-FS, defined as the time from the first day of SBRT to the start of palliative ADT). The main endpoints in oligo-CRPC were DPFS and second-line systemic treatment-free survival (STFS, defined as the time between the first day of SBRT to the start of systemic therapies such as Abiraterone, Enzalutamide, or Docetaxel). The Common Terminology Criteria for Adverse Events (CTCAE) v 4.0 were used to grade the toxicity of radiation measured from the final day of SBRT and censored at the time of disease development or subsequent treatment. Local failure, regional failure, or metastatic progression are all examples of progression. The OS endpoint was failure if a patient died before the end of follow-up, regardless of the reason of death or the date of the final follow-up.

### Statistical analysis

Quantitative factors were reported using the median and range, and qualitative variables were presented using frequency and percentage. SPSS 20.0 software was used for statistical analysis (SPSS for Window, IBM Corp., Armonk, NY, USA). Kaplan–Meier analysis was used to determine actuarial rates of LC, progression-free survival (PFS), and overall survival (OS). The time to event was defined as the interval between the date of diagnosis or SBRT, respectively, and the occurrence of the first clinical or radiological finding suggestive of disease recurrence.

The correlation between patient-related characteristics and treatment outcome was determined using log-rank testing. Pearson correlations between parameters were determined. Statistical significance was defined as a *p* value of ≤ 0.05.

## Results

### Characteristics of patients

In terms of 68 Ga-PSMA-PET/CT-directed radical irradiation of all lesions for BCR following surgery or definitive radiotherapy, 44 patients met the inclusion criteria (Table 1). Additionally, following a 68 Ga-PSMA-PET/CT-restaging for BCR, six patients underwent another course of SBRT/IGRT for newly diagnosed oligorecurrent metastases and one patient underwent two courses of SBRT.

**Table 1:** Characteristics of the patients

Patients		Values	Range	%
		44 pts		
Age at first diagnosis	Mean	60.5 yrs	47–73	n.a
	Median	60.9 yrs		n.a
Age at biochemical relapse	Mean	66.5 yrs	48–82	n.a
	Median	68 yrs		n.a
PSA at first diagnosis	Mean	30.27 ng/mL	5.2–448	n.a
	Median	11 ng/mL		n.a
	PSA > 20 ng/ml	19 pts		
PSA nadir after primary treatment	Mean	2.37 ng/mL	0.006–44	n.a
	Median	0.49 ng/mL		N.a
Gleason score	7	26 pts		(59%)
	≥ 8	18 pts		(41%)
Risk factors	pT3 a–b	28 pts		(64%)
	pN1	8 pts		(18%)
	R1	10 pts		(23%)
	D’Amico risk classification @ first diagnosis (*n*)			
	Intermediate	23 pts		52.3%
	High	21 pts		47.7%
Primary treatment	Radical radiotherapy	0 pts		
	Surgery (RPVE)	44 pts		100%
Type of oligometastatic	Metachronous	80 les		100%
	Synchronous	0 les		0%
	Metachronous oligorecurrence	64/80 les		
	Metachronous oligoprogression	14/80 les		
Interval between first diagnosis and biochemical relapse (years)	Mean	5.6 yrs	0.33 -19	n.a
	Median	4 yrs		n.a
Treatment courses		52		100%
2nd course		7		
3rd course		1		
Treated lesions		80 les		100%
Lymphatic nodes (*n* = 56)	Obturator	17		
	Iliac	18		
	Pararectal	14		
	paraaortic	7		
Bone metastasis (*n* = 24)	Pelvis	6		
	Vertebra	7		
	Rib	9		
Single lesion		14		
RT History^a^		27/44		
Prior RT of prostatic fossa		27		
ADT with first RT		5/44		
Use of ADT	Yes @ 1st course of SBRT	3		
	Yes @ 2nd course of SBRT	0		
	No	41		
Follow-up (months)	Mean	18	Range 3–42	n.a
	Median			n.a
GTV_Volume (mL)	Mean	4.84	0.22–20.69	n.a
	Median	3.8		n.a
PTV_Volume (mL)	Mean	27.82	3.3–88	n.a
	Median	25		n.a
SIB^b^	31/44			

*n.a.* not applicable

^a^Radiotherapy at first diagnosis

^b^13 patients had no elective treatment volumes

**Fig. 2:**
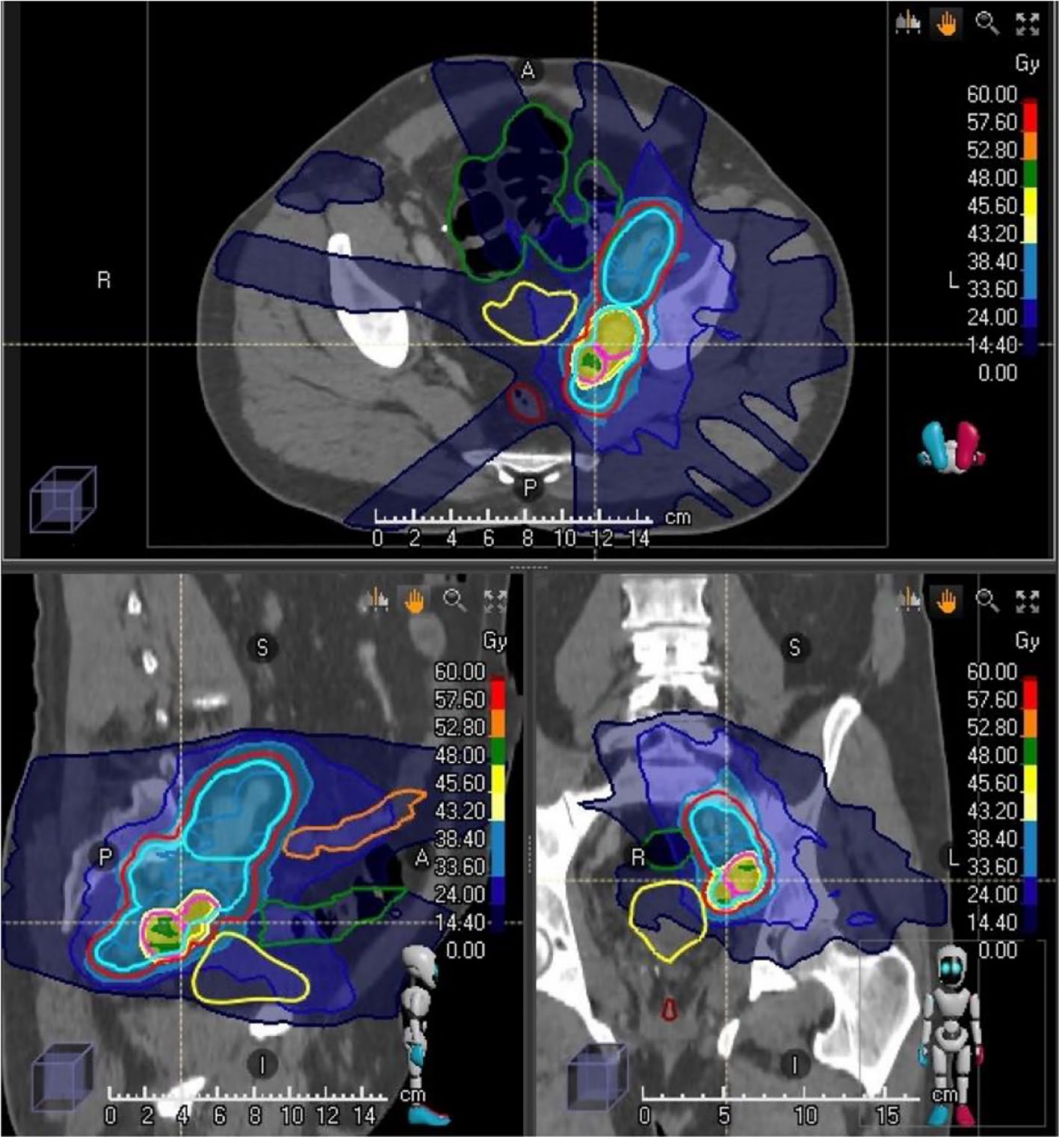
Radiation dose distribution of high-dose treatment of the PSMA-PET-positive nodal volume at ablative dose in 12 fractions as simultaneous integrated boost within elective dose to the entire lymphatic region. Dose in Gray as color-coded on the right-hand scale

**Fig. 3:**
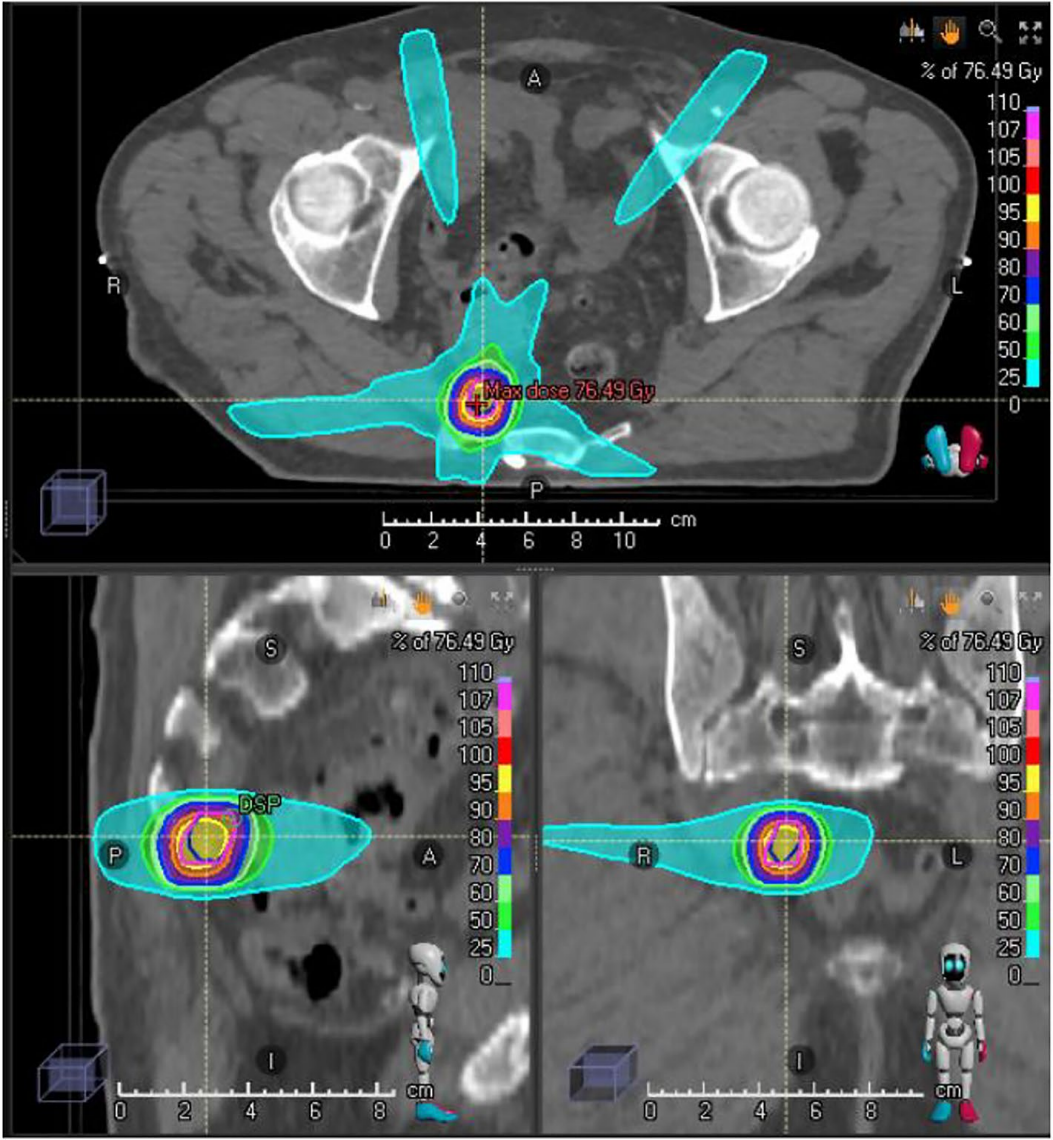
Radiation dose distribution of high-dose treatment of the PSMA-PET-positive nodal volume at ablative dose in 12 fractions without treatment of an elective nodal volume because of prior pelvic radiotherapy. Dose in gray as color-coded on the right-hand scale

Overall, 52 treatments (44 patients) of 80 oligometastases (total of 56 lymph-node metastases and 24 bone metastases) were conducted, together with one simultaneously discovered local recurrence (LR) (80 lesions). Solitary metastases were found in 19 patients (43%). (Table 1 patients’ characters). SBRT, defined as by Guckenberger et al. (2020), in 1–12 fractions was employed in 69% of the patients and hypofractionation in 31% (Table 2) (Figs. 2, 3).

**Table 2:** Radiotherapy dose regimes

Dose regime		BED_2_	BED_3_	BED_10_	EQD2_2_	EQD2_1,5_	EQD2_3_	EQD2_10_
48/4 Gy	20/52 (38%)	144	112	67.2	72	75	67.2	56
60/3 Gy	16/52 (31%)	150	120	78	75	77.14	72	65
35/7 Gy	8/52 (15%)	157.5	116.67	59.5	78.75	85	70	49.58
30/ 6 Gy	2/52 (4%)	105	80	45	52	64.3	48	37.5
40/5 Gy	2/52 (4%)	140	106.6	60	70	74.3	64	50
40/4 Gy	4/52 (8%)	144	112	67.2	60	62.86	56	46.7

The median age at the time of the initial diagnosis was 64.4 years (range 52–76 years) and the mean ECOG performance status was 0 (range: 0–2). Regarding pTNM, the majority of patients had at least one high-risk factor (pT3a: 15; pT3b: 13; pN1: 7; R1:10. Twenty-seven patients (61%) had a history of previous irradiation of the prostatic fossa in salvage or adjuvant settings (total dose: 66.0–74.0 Gy) 27.8 months (median; range 6–48 months) following RPVE.

All patients had 68 Ga-PSMA-PET/CT imaging for BCR, which was triggered by at least two PSA spikes of 0.2 ng/mL or above. At the time of imaging, the average PSA level was 1.8 ng/mL (range: 0.23– 33). Eighty lesions positive for 68 Ga-PSMA were discovered in PET/CT at a mean of 66 months (range 5–228 months) after RPVE. The majority of nodal oligometastases were found in the iliac and perirectal regions. The majority of bone metastases were seen in the pelvic bones.

Follow-up was 21 months on average (range, 3–42) and with a median of 19. Prior to SBRT, the median PSA level was 1.82 ng/dL, mean 4,067 (range, 0.23–33).

Mean size of the PSMA-GTV was 4.8 mL (median 3.8; range: 0.22–20.69) and mean PTV was 27.8 mL (median: 25; range: 3.3–88). A total of 31 treatments were with SIB-concept adding an elective treatment of the nodal region to an EQD2_2_ of 50 Gy.

Mean dose was 44.75 Gy (median 42; range 35–60) in a mean of 11.7 fractions (median, 12 range 5–20), with a mean daily dose of 4.37 Gy (median 4; range 3–8 Gy). Mean BED_2_ was 144.62 Gy (range 140–150), mean EQD2_3_ was 68.42 Gy, and mean EQD2_10_ was 56.923 Gy, while mean EQD2_2_ was 72,3.

### Outcome

**Fig. 4:**
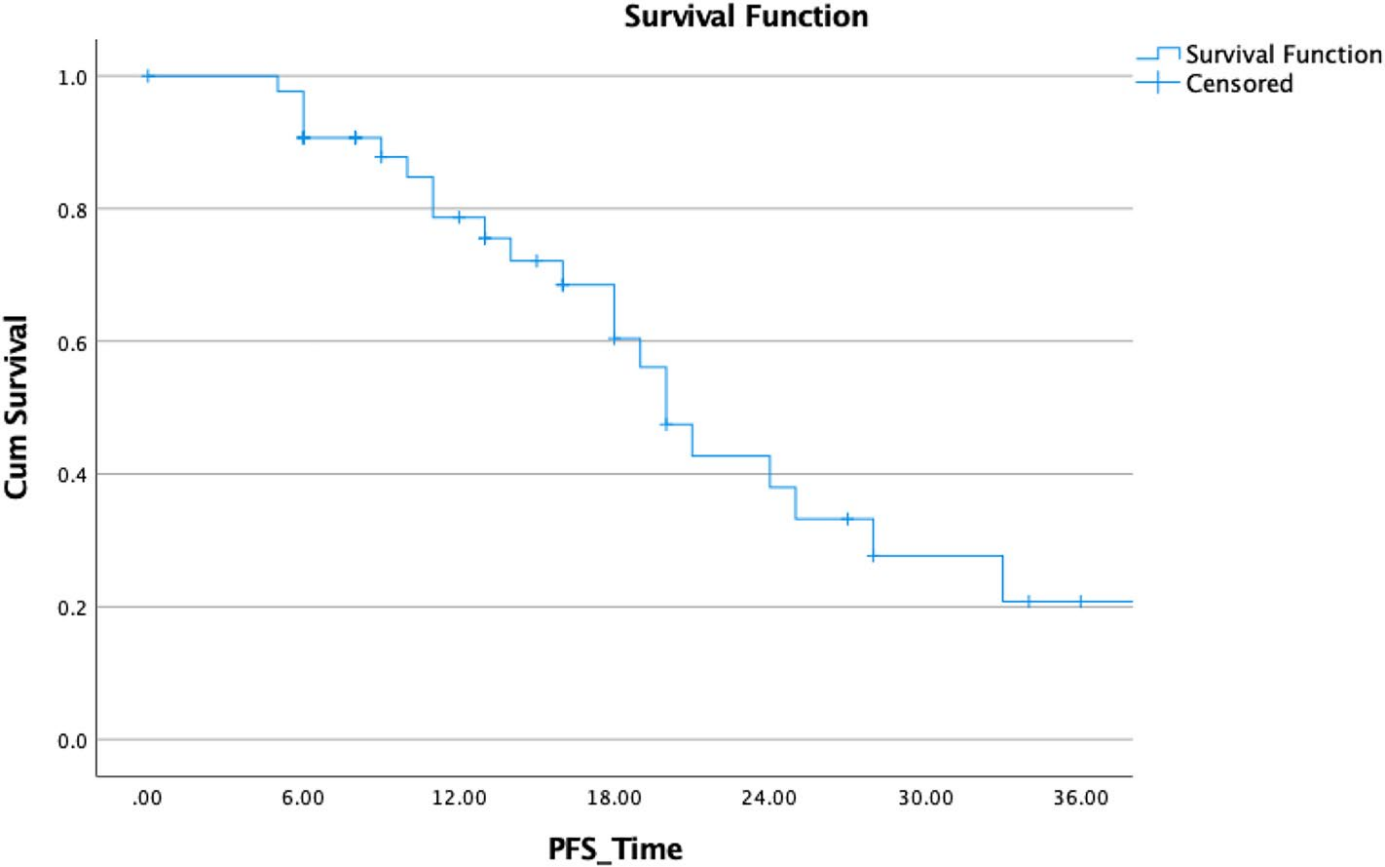
Biochemical progression-free survival in 44 patients with oligometastatic prostate cancer after SBRT or hypofractionated radiotherapy

In terms of effectiveness, the median PSA-nadir level following the completion of RT was 0.49 ng/mL. In 44 individuals, biochemical relapse, defined as a rise of PSA after reaching the nadir 3 times in a row or above 2 ng/mL or positive PSMA-PET lesion, occurred after an average of 16 months (range: 3–39) and median of 14 months. At the time of relapse, the median PSA level was 1.89 ng/mL (range: 0.8–11.57) (Figs. 1, 4).

**Fig. 5:**
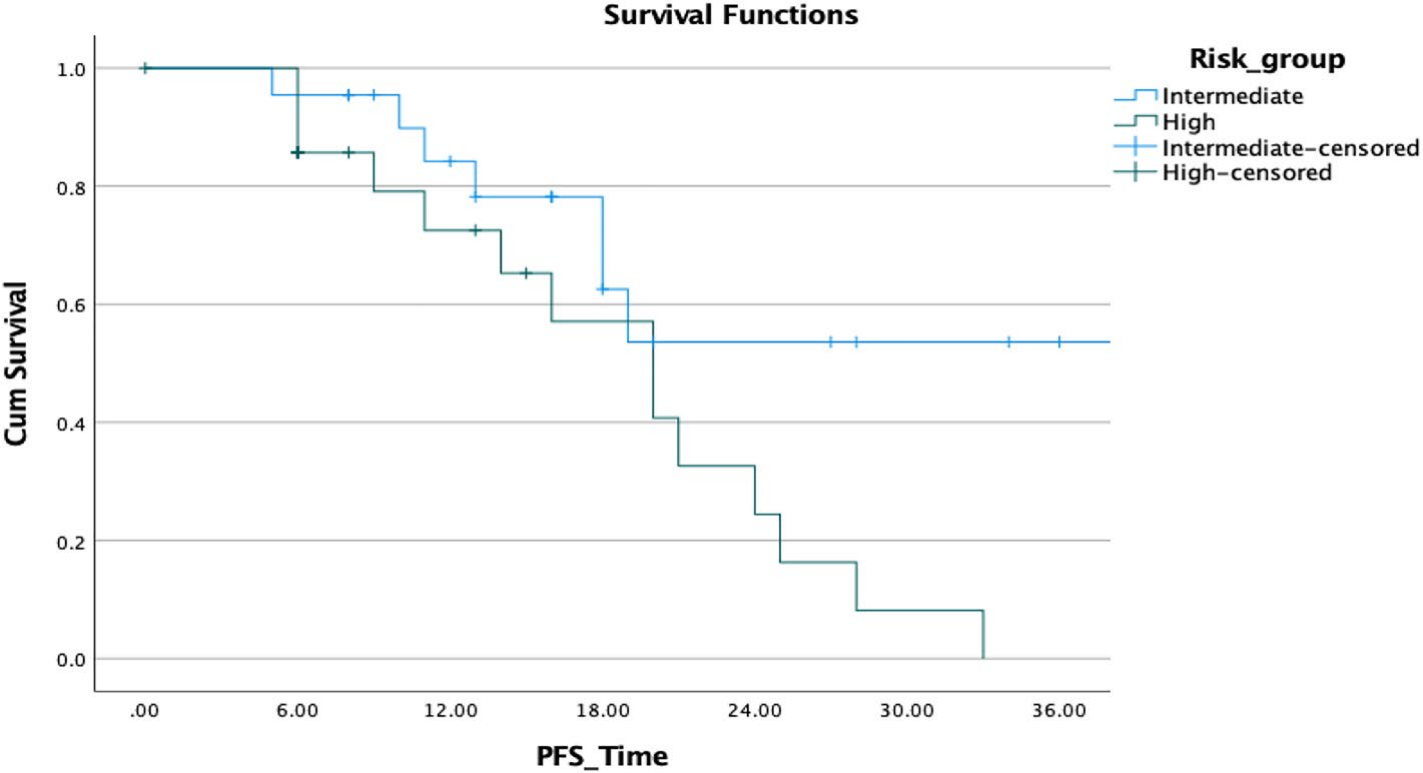
Comparison of biochemical PFS in patients with intermediate and high risk at the initial diagnose

Twenty of the 44 patients achieved biochemical control following the first SBRT, whereas 24 individuals developed PSA recurrence out of field. Following the first SBRT, three of these patients suffered a failure in the prostate bed. These patients had the following characteristics at first diagnosis: 1 intermediate and 2 high-risk D’Amico group, 1 with R1 resection, 1 with Pn1 category, all of them post RPVE and intervals between first therapy and biochemical relapse of 34, 46 and 18 months. None of them was treated with radiotherapy at first diagnosis. Two of them were treated with salvage EBRT and, subsequently, achieved complete PSA control. The third underwent salvage hypofractioned radiotherapy to the PSMA lesion in the prostatic fossa as he experienced a salvage EBRT 3 years prior to biochemical failure (Fig. 5).

Only 4 patients underwent a second course of SBRT, and all of them achieved a second biochemical control. Eleven patients received ADT after a median of 17 months after SBRT (range 6–33 Months). Only 5/44 Patients underwent SBRT under ADT. Three of them experienced biochemical failure after a median of 18 months after SBRT. Two of those who developed biochemical failure received chemotherapy and one patient was a candidate for Lutetium radio-ligand therapy. Three of 44 patients showed biochemical failure, without any PSMA + ve lesions, they underwent wait and see strategy.

### Patterns of failure

**Fig. 6:**
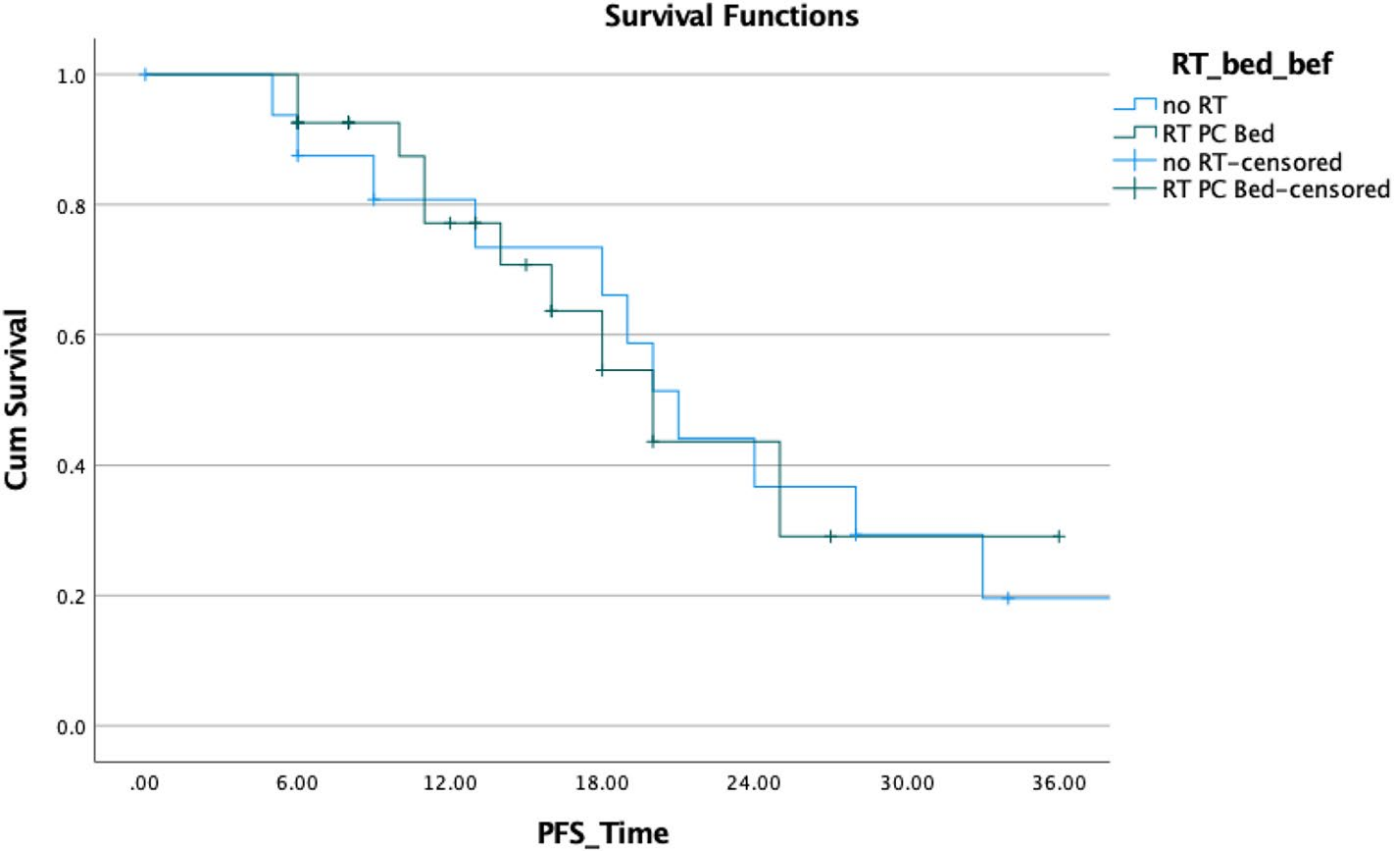
PFS of 44 patients post-radical prostatovesculectomy with (*n* = 27) and without (*n* = 17) adjuvant or salvage prostatic fossa radiotherapy

The following pattern of relapse emerged in patients who suffered a PSA recurrence following SBRT treatment (Table 3). Three patients demonstrated disease progression in the prostatic bed (treated with salvage EBRT). Recurrence in lymph nodes occurred in nine individuals, but only three patients were eligible for SBRT: six patients had recurrent disease and were treated with systemic treatment (all treated with ADT), because they were identified as having disseminated disease (Fig. 6).

**Table 3:** Outcome after first series of SBRT/hypofractionated radiotherapy

	Number of patients	%
Biochemical complete response^a^ CR	20	45.5
BCR without PSMA-positive lesion	4	9.1
In field recurrence	0	0%
Out of field nodal	10	22.7
Out of field bony	7	15.9
Prostatic bed recurrence post SBRT hypofractionation at distance from the prostatovesicular region	3	6.8

^a^Definition of biochemical complete response was biochemical relapse, defined as a rise of PSA after reaching the nadir 3 times in a row or above 2 ng/mL or positive PSMA-PET lesion

No recurrences were observed in lesions that were treated by SBRT or hypofractionated radiotherapy. It is worth noting that three out of nine patients with nodal recurrence and oligometastatic disease relapsed in a node adjacent to previously treated lymph nodes but out of radiation field. Patients with bone metastases treated with SBRT who relapsed did not demonstrate any spread in the immediate vicinity of the treated lesion: they developed new distant metastasis in other bone segments.

### Toxicity

All patients received the full course of treatment, which was usually well tolerated with a minor pattern of toxicity. In the acute context, 42/44 individuals experienced Grade 1–2 side effects. The most often reported effects were nausea and fatigue. During follow-up, only two patients reported > grad 3e gastrointestinal (GI)-Toxicity, one suffered from GI grade 3 toxicity, and these resolved with medications. GI grade 4 toxicity occurred in only one patient, who had two lymph nodes (iliacal and pararectal) and who was treated with 12 fractions rather than fewer fractions to reduce bowel toxicity, Despite rectal and colon dose within the constrains; the patient developed a colon perforation. This occurred potentially due to increased bowel sensibility as suggested by a history of spontaneous perforation 2 years before radiotherapy by chronic diverticulosis. Another patient was reported to have grade 3 Genitourinal (GU) toxicity. Geriatric evaluations revealed no deterioration in health status during the length of the follow-up.

**Fig. 7:**
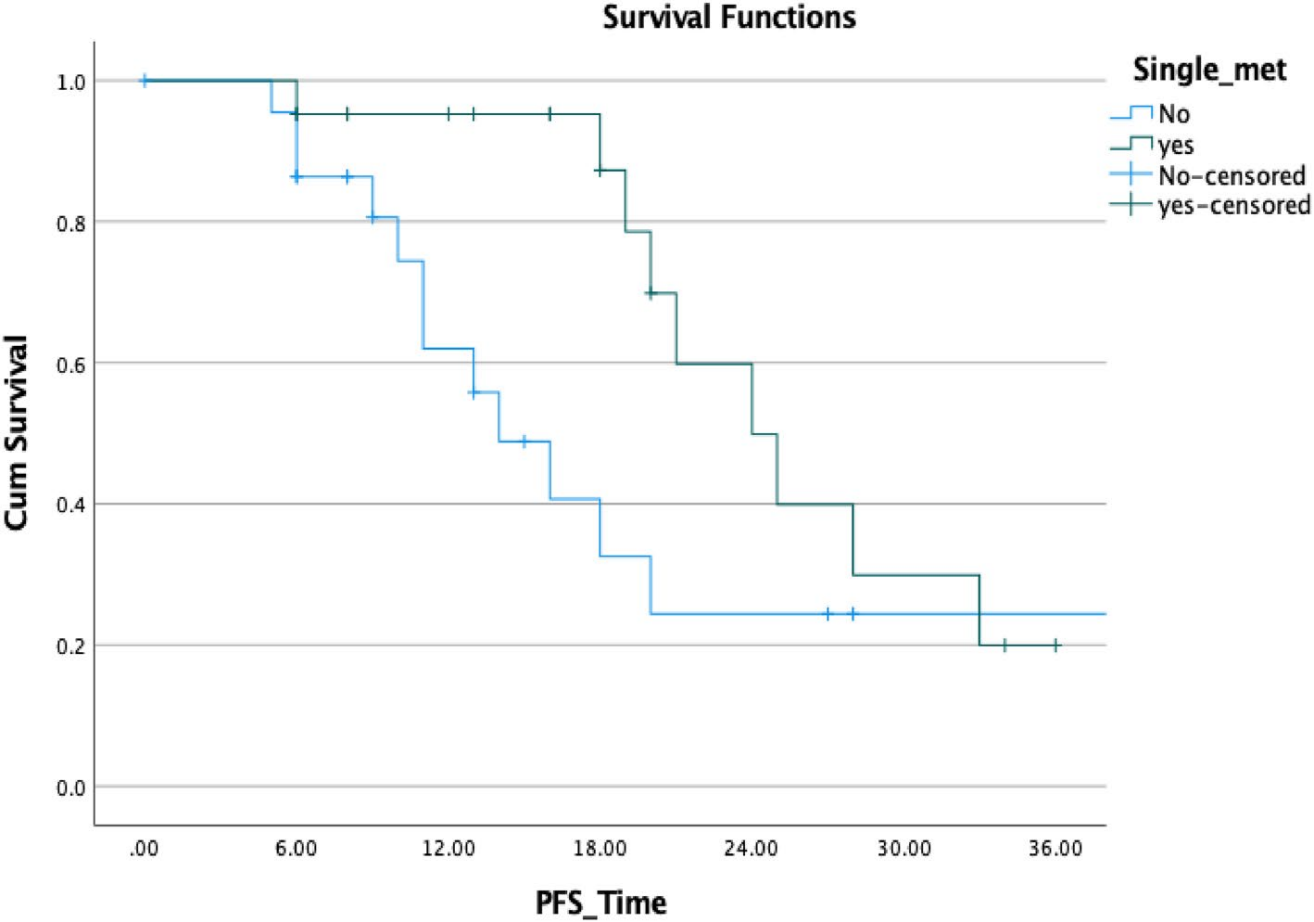
Comparison of biochemical PFS in patients with solitary lesions and with > 1 lesion

**Fig. 8:**
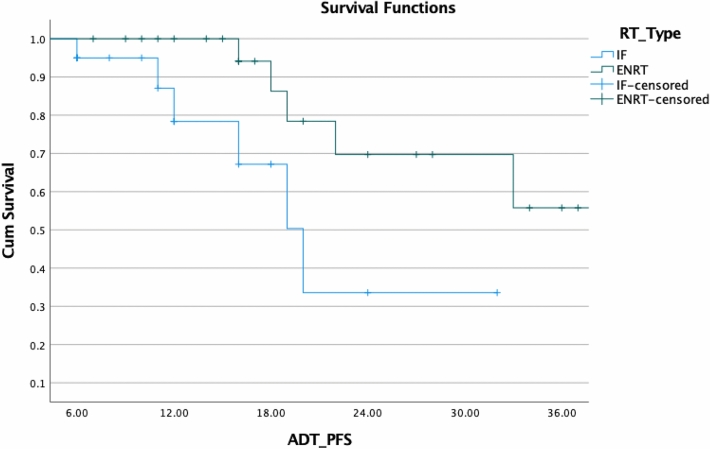
Comparison of ADT-free_biochemical PFS with ENRT and IF

Finally, we conducted uni- and multivariate analyses for biochemical control of patients with oligometastatic disease (Table 4). Improved BCR-PFS was not linked to a history of previous radiotherapy of the prostatic fossa, Gleason score, loco-regional radiotherapy, PSA nadir, and age in our analyses using the log-rank statistic and Kaplan–Meier estimates (*p* = 0.505, 0.903, 0. 602, 0.091, and 0.354, respectively). Although not statistically significant, there was a tendency toward improved survival in patients with a single lesion versus those with more than one lesion (*p* = 0.405; HR: 0.605); however, Kaplan–Meier demonstrated a superior PFS in the first 2 years (Fig. 7). A decrease in PSA following SBRT was not associated with an improved prognosis for OS. The only two variables associated with improved PFS, and ADT-PFS were baseline PSA prior SBRT and longer intervals between RPVE and the BCF (*p* = 0.004 and 0.007, HR: 1.010 and 0.752, respectively) (Fig. 8).

**Table 4:** Univariate Cox regression model results for biochemical progression-free survival all of patients surviving > 3 months after SBRT

	*p* value Sig	Hazard ratio	Lower CI or SD?	Upper
Initial PSA	**0.004**	1.010	1.003	1.017
RT of the prostatic bed	0.505	1.456	0.482	4.397
Single lesion	0.405	0.605	0.183	1.976
GS	0.903	1.048	0.493	2.229
ENRT	0.602	1.373	0.417	4.524
Interval1st D to BCR before SBRT	**0.007**	0.752	0.610	0.926
PSA-Nadir after primary treatment	0.092	0.562	0.288	1.098
Age at first diagnosis	0.354	1.033	0.964	1.108

Statiscally significant *p* values are in bold

## Discussion

To the best of our knowledge, this is the first published study to report a technique combining ENRT with a boost to the IF volume using either SBRT or hypofractionation. All other published studies on this combination have used conventional fractionation (CF) schedules (Ost et al. 2016; Bleser et al. 2017; Phillips et al. 2020; Koerber et al. 2020; Fodor et al. 2017; Würschmidt et al. 2011; Lépinoy et al. 2019; Kirste et al. 2021; Decaestecker et al. 2014).

In this retrospective analysis, we describe our experience with patients who received Linac-based SBRT-IMRT for oligometastatic prostate cancer. In our experience, patients with an oligometastatic status who underwent SBRT or ENRT with a boost to the IF volume had excellent local control. Indeed, all the treated lesions were locally controlled in our analysis after 1 and 2 years. This finding corroborates Palma DA et al., indicating that targeted treatment, such as SBRT, is an effective method of disease management in patients with four or fewer metastatic lesions (Palma et al. 2012). Additionally, dose administration using daily cone-beam CT (CBCT) conducted prior to each session of RT ensures adherence to dose restrictions for organs at risk and achievement of prescription to the target, hence limiting the pattern of toxicity. In our cohort, we experienced no relevant differences in the toxicities between the two techniques. Nevertheless, one patient, who underwent ENRT, suffered a grade 4 GI toxicity which was attributed to a previous history of perforation by known diverticulosis. This occurred despite respecting the dose constraints to the OARs. However, it might still serve as a warning sign for the GI tolerance when employing hypofractioned regimes (Fig. 5; Table 5).

**Table 5:** Review the published results of SBRT in oligometastatic Prostata cancer

	No of patients/primary/oligometastatic State	Primary endpoint	Median PFS (months)	Toxicity (≥ G3)
Ost et al. (2018)	62 Oligorecurrent	ADT-free survival	21 versus 13	0% versus 0%
Bowden et al. (2020)	199 Oligorecurrent	Treatment escalation	27	0%
Siva et al. (2018) and	33 Oligorecurrent	Feasibility	24	3%
Deek et al. (2021)	68Oligoprogressive	PFS	10	0%
Our study	44 (80 Lesions) oligorecurrent	PFS	19	4%

In our study, we treated 13 patients using a focal strategy by targeting only the lesion (IFRT), while in 31/44, we used elective radiation technique with SIB (ENRT). The analysis did not show differences in PFS or ADT-free survival. There is a lack of comparative evidence for IF methods vs elective RT. A total of 62 patients with nodal oligorecurrent PC were treated with either elective nodal RT or involved node SBRT and analyzed in a study by Lepinoy et al. for outcome and toxicity (Lépinoy et al. 2019). Twenty-seven patients suffered biochemical failure after a median follow-up of 41.8 months (5.9–108.1 months), with 23 in the IFRT group and 4 in the ENRT group. The median 3-year overall failure rate of 69.5 percent (95 percent CI 56.0–79.6 percent). The median 3-year-freedom from failure was improved with s-EFRT: in the IFRT group, it was 55.3 percent (95 percent CI 37.0–70.3 percent), and in the ENRT group, it was 88.3 percent (95 percent CI 66.9–96.1%; *p* = 0.0094). Late gastrointestinal and urinary tract toxicities were not significantly different between groups (*p* = 0.576 for both).

Similar findings of reduced nodal recurrences were made by De Bleser et al. (2017). They observed a significantly higher rate of late toxicities with ENRT compared to IFRT (16% in 130 patients vs 5% in 202 patients) including 3 patients with grade 3 GU and 1 patient with grade 4 combined GI/GU toxicities. Finally, a multicenter report of 204 patients with IF versus 190 patients with ENRT found significantly improved 3-year bPFS in the latter group with 61% versus 22%. Again, higher toxicity was reported with ENRT at 0.8% and 3% for acute and late grade ≥ 3 versus none in the IF group.

Another German study by Kriste et al. showed PSMA directed radiotherapy plus elective RT was associated with a significantly improved 3-year bRFS compared to PSMA directed radiotherapy alone (53 vs. 37%; *p* = 0.001; Kirste et al. 2021). This research looked primarily at elective RT of the prostate bed. Patients who received just ENRT progressed considerably more often and had a lower 3-year bRFS (22%) than patients who received both ENRT and elective prostate bed RT (ePBRT) (3-year bRFS 61 percent; *p* 0.001). The majority of patients (52.0%) were treated with conventionally fractionated RT or conventionally fractionated RT with a simultaneous integrated boost (SIB) approach 130. (33.0 percent). SBRT was employed in 38 (9.6%) of the patients, while combination of SBRT and conventional RT was used in 21 (5.4%) of the patients.

These comparisons support the hypothesis that invisible microscopical nodal metastases in the vicinity of the PET-positive nodes lead to recurrent disease. In the light of these studies with CF therapy, the current study agrees with these results with respect to regional control and to moderately increased toxicity, however, with shorter treatment duration for ENRT.

SBRT was effective in controlling tumor regardless of the location of recurrence, with response rates nearly comparable in cases of nodal disease and bone metastases. In patients with nodal disease who relapsed following RT and continued to have an oligometastatic pattern of disease, failure invariably occurred in nodes next to previously treated lymph nodes, similar to what Decaester et al. reported (2014). We hypothesize that this finding is significant, because it may be utilized to alter the SBRT method in patients with nodal metastases by delivering a fractionated radiation dosage to the affected chain's lymph nodes. Because imaging methods identify micrometastatic illness infrequently, the disease burden in the nearest lymph nodes is underestimated (Tilki et al. 2013) (Fig. 2).

Pasqualetti F et al. evaluated the clinical results of 29 patients with oligometastatic PCa treated with [18F] FMCHPET/CT-guided SBRT in prospective research, and there was a substantial influence on patient treatment management (Pasqualetti et al. 2016). It achieved disease control, as measured by PSA levels, in 20 individuals, avoiding the requirement of systemic medication. In the remaining nine patients, he postponed the start of systemic medication for 36 months. For SBRT, it was discovered that the higher the EQD2 (equivalent dose in 2-Gy fractions) was, the better the overall survival rate (BPFS) was determined to be by Schick et al. (HR 0.37, *p* = 0.034; Zacho et al. 2018).

Ost et al. (2016) investigated the pattern of relapse following SBRT for nodal recurrent PCa. They studied 72 patients treated for 89 lymph-node metastases and discovered additional metastases in 68% of instances. They evaluated 72 patients treated for 89 lymph-node metastases and discovered that 68% of newly metastases occurred outside the RT-Field, with a median distant-PFS of 21 months. According to the authors, 88 percent of patients had more than three metastases at the time of BCR. They reported that 14% of patients treated with s-IFRT experienced grade 1 toxicity, 3% had grade 2 toxicity, and none had grade 3 or greater toxicity in large multicenter retrospective research.

Another German group led by Habl et al. (2017) conducted a retrospective study on a limited number of patients (15 patients) with oligometastatic prostate cancer who had no more than two bone metastases and were treated with curative SBRT (Biological Effective Dose-BED > 100 Gy) to the metastatic sites. They reported that the median b-RFS was 6.9 months (range: 1.1–28.4 months), and the 2-year LRFS was 100%. They demonstrated that curative SBRT for metastatic disease is safe and has a high rate of local control. Similarly, Jereczek-Fossa et al. (2017) used SBRT to treat 94 patients with nodal oligometastatic disease, administering a median total dose of 24 Gy (range 15–36 Gy) in three fractions (median BED 152 Gy). The majority of patients (94.7%) had one or two metastatic nodes.

Our study demonstrates that postoperative RT can eliminate LR (local recurrence). In our cohort, from 17 patients who have no history of irradiation of prostatic fossa, 3 patients (17.6%) developed local prostate bed recurrence after ablative RT for oligometastases. That is also evidenced by the low rate of LR in patients treated with RPVE + RT compared to the RPVE-only group by Sobol et al. (2017). mpMRI and Choline PET were utilized in this work to physically map early patterns in 223 participants with BCR among a particularly pure cohort of post-RP patients who had not received any radiation or ADT treatment. The findings revealed that most varieties of relapse are neither as localized nor as broadly metastatic as often assumed, but rather a mix of local and/or regionally metastatic recurrences contained inside the pelvis. This mapping investigation provided additional evidence for the expansion of salvage RT fields into the pelvis as reviewed in RTOG 0534. Assuming total disease eradication by salvage RT, a cure response might be expected in 33% of men treated with salvage fossa irradiation alone. Extending the sRT area to include pelvic lymph nodes distal to the common iliac bifurcation may increase curative responses closer to 50%.

Also, another study by Rischke et al. provides more evidence for the underestimation of nodal disease in PET/CT (Rischke et al. 2015). Patients in this research received extra RT following PET/CT-guided salvage lymph-node resection. The addition of RT to the areas with PSMA-expressing tumors on PET/CT improved 5-year PFS from 26.3 to 70.7 percent, demonstrating residual micrometastasis following surgery. In a similar manner to nodal illness, underestimating of subclinical, microscopic disease is likely to occur in the prostate bed, which has the highest risk of microscopic disease following radical prostatectomy. In addition to the poor spatial resolution of PET/CT, tracer excretion via the bladder, with subsequent blurring of the region of the prostatic fossa, makes detecting a local recurrence in the prostate fossa challenging. Our data suggest that RT to the prostate bed can be omitted and done as salvage if required to avoid unnecessary GU/GI toxicity.

Five of our 44 participants underwent ADT concurrently. Due to the uncertain timing of ADT initiation within the patient population, the PSA-PFS findings should be evaluated cautiously. In a prospective single-arm Spanish-Swiss research (Schick et al. 2013), the combination of locally ablative RT and concurrent ADT resulted in a PFS of 54%, a clinical PFS of 58 percent, and an OS of 92% after a median time period of 30 months. ADT was not administered in a systematic manner and was administered for a median of 18 months (range, 3–34 months).

Due to the retrospective and monocentric nature of our investigation, there are significant limitations. Additionally, because the median duration of follow-up is relatively short, the essential endpoints of overall survival and prostate cancer-specific survival could not be examined. The majority of patients underwent pretreatment imaging with PSMA-PET/CT, which was proven to have a high sensitivity for identifying prostate cancer metastases (Zacho et al. 2018). However, due to the absence of histologic confirmation, we were unable to rule out false-positive and false-negative PSMA-PET lesions. Concurrent ADT administration was inconsistent, complicating the assessment of PSA kinetics and bPFS.

However, in the absence of further prospective data, this study throws some insight on the hitherto underrepresented population of patients with bone oligometastatic prostate cancer treated with MDT and classified according to current ESTRO/EORTC oligometastatic disease subclassifications. Although hormone sensitivity and the oligometastatic disease category were no longer significant predictors of oncological outcome in the multivariate analysis, the large differences in oncological outcome between the groups underscore the need for a more precise classification of patients with bone oligometastatic disease (Fig. 3).

## Conclusion

In conclusion, our findings show that PSMA-PET/CT-guided stereotactic and hypofractionated radiotherapy with elective nodal therapy may be a viable therapeutic option for OMPC patients, including those with recurrent oligometastatic disease. The hypofractioned ENRT in 12 fractions looks to be safe and feasible, and not associated with high toxicity. The beginning or escalation of palliative ADT, as well as its possible adverse effects, can be prevented in this manner. SBRT-treated metastases had high local control rates with low toxicity. Elongated PFS is predicted by low PSA levels. Randomized trials are required to validate our findings.

## Abbreviations

RP: Radical prostatectomy
RT: Radiotherapy
SBRT: Stereotactic body radiation therapy
OMD: Oligometastic disease
ADT: Androgen deprivation therapy
ECOG: Eastern Cooperative Oncology Group
OS: Overall Survival
PFS: Progression-free survival
BCF: Biochemical Failure
IGRT: Image-guided radiation therapy
PCa: Prostata cancer
SIB: Simultaneous integrated boost
HoFRT: Hypofractionated radiotherapy
bRFS: Biochemical relapse free
ENRT: Elective nodal radiotherapy
IF: Involved field
BCR: Biochemical recurrence
rcN1: Oligorecurrent nodal disease
M1: Oligorecurrent metastatic disease

## References

[CR1] Cheung P (2016). Stereotactic body radiotherapy for oligoprogressive cancer [serial online]. Br J Radiol.

[CR2] De Bleser E, Tran PT, Ost P (2017). Radiotherapy as metastasis-directed therapy for oligometastatic prostate cancer. Curr Opin Urol.

[CR300] Bowden P, See AW, Frydenberg M, Haxhimolla H, Costello AJ, Moon D, Ruljancich P, Grummet J, Crosthwaite A, Pranavan G (2020). Fractionated stereotactic body radiotherapy for up to five prostate cancer oligometastases: Interim outcomes of a prospective clinical trial. Int. J. Cancer.

[CR3] Decaestecker K, De Meerleer G, Lambert B, Delrue L, Fonteyne V, Claeys T (2014). Repeated stereotactic body radiotherapy for oligometastatic prostate cancer recurrence. Radiat Oncol.

[CR4] Deek MP, Taparra K, Phillips R, Velho PI, Gao RW, Deville C, Song DY, Greco S, Carducci M, Eisenberger M, DeWeese TL, Denmeade S, Pienta K, Paller CJ, Antonarakis ES, Olivier KR, Park SS, Tran PT, Stish BJ (2021). Metastasis-directed therapy prolongs efficacy of systemic therapy and improves clinical outcomes in oligoprogressive castration-resistant prostate cancer. Eur Urol Oncol.

[CR5] Eisenhauer E, Therasse P, Bogaerts J, Schwartz LH, Sargent D, Ford R (2009). New response evaluation criteria in solid tumors: revised RECIST guideline (version1.1). Eur J Cancer.

[CR6] Fodor A, Berardi G, Fiorino C, Picchio M, Busnardo E, Kirienko M (2017). Toxicity and efficacy of salvage carbon 11-choline positron emission tomography/computed tomography-guided radiation therapy in patients with lymph node recurrence of prostate cancer. BJU Int.

[CR7] Franzese C, Zucali PA, Di Brina L (2018). The efficacy of stereotactic body radiation therapy and impact of systemic treatments in oligometastatic patients from prostate cancer. Cancer Med.

[CR8] Guckenberger M, Baus WW, Blanck O, Combs SE, Debus J, Engenhart-Cabillic R, Gauer T, Grosu AL, Schmitt D, Tanadini-Lang S, Moustakis C (2020). Definition and quality requirements for stereotactic radiotherapy: consensus statement from the DEGRO/DGMP Working Group Stereotactic Radiotherapy and Radiosurgery. Strahlenther Onkol.

[CR9] Habl G (2017). Oligometastases from prostate cancer: local treatment with stereotactic body radiotherapy (SBRT). BMC Cancer.

[CR10] Jereczek-Fossa BA (2017). Salvage stereotactic body radiotherapy for isolated lymph node recurrent prostate cancer: single institution series of 94 consecutive patients and 124 lymph nodes. Clin Genitourin Cancer.

[CR11] Kirste S, Kroeze S, Henkenberens C, Schmidt-Hegemann NS, Vogel M, Becker J, Zamboglou C, Burger I, Derlin T, Bartenstein P, Ruf J, la Fougère C, Eiber M, Christiansen H, Combs SE, Müller AC, Belka C, Guckenberger M, Grosu AL (2021). Combining ^68^Ga-PSMA-PET/CT-directed and elective radiation therapy improves outcome in oligorecurrent prostate cancer: a retrospective multicenter study. Front Oncol.

[CR12] Kneebone A, Hruby G, Ainsworth H (2018). Stereotactic body radiotherapy for oligometastatic prostate cancer detected via prostate-specific membrane antigen positron emission tomography. Eur Urol Oncol.

[CR13] Koerber SA, Katharina S, Clemens K, Erik W, Matthias FH, Sonja K (2020). Clinical outcome of PSMA-guided radiotherapy for patients with oligor-ecurrent prostate cancer. Eur J Nucl Med Mol Imaging.

[CR14] Lépinoy A, Silva YE, Martin E, Bertaut A, Quivrin M, Aubignac L (2019). Salvage extended field or involved field nodal irradiation in 18F-fluorocholine PET/CT oligorecurrent nodal failures from prostate cancer. Eur J Nucl Med Mol Imaging.

[CR15] Lievens Y, Guckenberger M, Gomez D, Hoyer M, Iyengar P, Kindts I, Méndez Romero A, Nevens D, Palma D, Park C, Ricardi U, Scorsetti M, Yu J, Woodward WA (2020). Defining oligometastatic disease from a radiation oncology perspective: An ESTRO-ASTRO consensus document. Radiother Oncol.

[CR16] Milano MT, Katz AW, Muhs AG, Philip A, Buchholz DJ, Schell MC, Okunieff P (2008). A prospective pilot study of curative-intent stereotactic body radiation therapy in patients with 5 or fewer oligometastatic lesions. Cancer.

[CR17] Ost P, Jereczek-Fossa BA, As NV (2016). Progression-free survival following stereotactic body radiotherapy for oligometastatic prostate cancer treatment-naive recurrence: a multi-institutional analysis. Eur Urol.

[CR18] Ost P, Reynders D, Decaestecker K (2018). Surveillance or metastasis-directed therapy for oligometastatic prostate cancer recurrence: a prospective, randomized, multicenter phase II trial. JCO.

[CR19] Palma DA, Haasbeek CJ, Rodrigues GB (2012). Stereotactic ablative radiotherapy for comprehensive treatment of oligometastatic tumors (SABRCOMET): study protocol for a randomized phase II trial. BMC Cancer.

[CR20] Pasqualetti F, Panichi M, Sainato A, Matteucci F, Galli L, Cocuzza P (2016). [(18)F]Choline PET/CT and stereotactic body radiotherapy on treatment decision making of oligometastatic prostate cancer patients: preliminary results. Radiat Oncol.

[CR21] Pembroke CA, Fortin B, Kopek N (2018). Comparison of survival and prognosis factors in patients treated with stereotactic body radiotherapy for oligometastases or oligoprogression. Radiother Oncol.

[CR22] Phillips R, Shi WY, Deek M, Radwan N, Lim SJ, Antonarakis ES (2020). Out- comes of observation vs stereotactic ablative radiation for oligometa-static prostate cancer: the ORIOLE phase 2 randomized clinical trial. JAMA Oncol.

[CR23] Rischke HC, Schultze-Seemann W, Wieser G, Krönig M, Drendel V, Stegmaier P (2015). Adjuvant radiotherapy after salvage lymph node dissection because of nodal relapse of prostate cancer versus salvage lymph node dissection only. Strahlenther Onkol.

[CR24] Schick U, Jorcano S, Nouet P, Rouzaud M, Vees H, Zilli T, Ratib O, Weber DC, Miralbell R (2013). Androgen deprivation and high-dose radiotherapy for oligometastatic prostate cancer patients with less than five regional and/or distant metastases. Acta Oncol.

[CR25] Siva S, Bressel M, Murphy DG (2018). Stereotactic Abative Body Radiotherapy (SABR) for oligometastatic prostate cancer: a prospective clinical trial. Eur Urol.

[CR26] Sobol I, Zaid HB, Haloi R (2017). Contemporary mapping of post-prostatectomy prostate cancer relapse with 11C-choline positron emission tomography and multiparametric magnetic resonance imaging. J Urol.

[CR27] Tilki D, Reich O, Graser A, Hacker M, Silchinger J, Becker AJ (2013). 18F-Fluoroethylcholine PET/CT identifies lymph node metastasis in patients with prostate-specific antigen failure after radical prostatectomy but underestimates its extent. Eur Urol.

[CR28] Tree AC, Khoo VS, Eeles RA (2013). Stereotactic body radiotherapy for oligometastases. Lancet Oncol.

[CR29] Triggiani L, Alongi F, Buglion M (2017). Efficacy of stereotactic body radiotherapy in oligorecurrent and in oligoprogressive prostate cancer: new evidence from a multicentric study. Br J Cancer.

[CR30] Würschmidt F, Petersen C, Wahl A, Dahle J, Kretschmer M (2011). [18F] fluoroethylcholine-PET/CT imaging for radiation treatment planning of recurrent and primary prostate cancer with dose escalation to PET/CTpositive lymph nodes. Radiat Oncol.

[CR31] Zacho HD, Nielsen JB, Haberkorn U, Stenholt L, Petersen LJ (2018). 68Ga-PSMA PET/CT for the detection of bone metastases in prostate cancer: a systematic review of the published literature. Clin Physiol Funct Imaging.

